# Realization and Technology Acceptance Test of a Wearable Cardiac Health Monitoring and Early Warning System with Multi-Channel MCGs and ECG

**DOI:** 10.3390/s18103538

**Published:** 2018-10-19

**Authors:** Wen-Yen Lin, Hong-Lin Ke, Wen-Cheng Chou, Po-Cheng Chang, Tsai-Hsuan Tsai, Ming-Yih Lee

**Affiliations:** 1 Department of Electrical Engineering, Center for Biomedical Engineering, Chang Gung University, Tao-Yuan 33302, Taiwan; wylin@mail.cgu.edu.tw (W.-Y.L.); k22060987@yahoo.com.tw (H.-L.K.); sito.19@gmail.com (W.-C.C.); 2 Division of Cardiology, Department of Internal Medicine, Chang Gung Memorial Hospital, Linkou, Tao-Yuan 33305, Taiwan; pccbrian@gmail.com (P.-C.C.); ttsai@mail.cgu.edu.tw (T.-H.T.); 3 Department of Industrial Design, Chang Gung University, Tao-Yuan 33302, Taiwan; 4 Graduate Institute of Medical Mechatronics, Center for Biomedical Engineering, Chang Gung University, Tao-Yuan 33302, Taiwan

**Keywords:** mechanocardiogram (MCG), smart clothes, heart failure (HF), left ventricular ejection fraction (LVEF), technology acceptance model (TAM)

## Abstract

In this work, a wearable smart clothing system for cardiac health monitoring with a multi-channel mechanocardiogram (MCG) has been developed to predict the myo-cardiac left ventricular ejection fraction (LVEF) function and to provide early risk warnings to the subjects. In this paper, the realization of the core of this system, i.e., the Cardiac Health Assessment and Monitoring Platform (CHAMP), with respect to its hardware, firmware, and wireless design features, is presented. The feature values from the CHAMP system have been correlated with myo-cardiac functions obtained from actual heart failure (HF) patients. The usability of this MCG-based cardiac health monitoring smart clothing system has also been evaluated with technology acceptance model (TAM) analysis and the results indicate that the subject shows a positive attitude toward using this wearable MCG-based cardiac health monitoring and early warning system.

## 1. Introduction

Mechanocardiogram (MCG) [1] or so-called Seismocardiogram (SCG) [2,3] was proposed in early 1990 using an inertial motion sensing device, i.e., an accelerometer, for cardiac activity monitoring. This technology is capable of identifying feature points of the cardiac activity events, such as the opening and closing of heart valves [4], as well as some heart systolic and diastolic characteristics, such as isovolumic movement (IM), isotonic contraction (IC), peak of rapid diastolic filling (RF), and peak of atrial systolic (AS) [5]. It has also turned into an emerging method for cardiac health monitoring as mature MEMS-based technology and the development of integrated circuit (IC) process [6].

Most of the related works employing MCG/SCG technologies used a single accelerometer placed on the sternum of the chest to record the mechanical composite vibration signal incurred from the complex heart beat activities within a cardiac cycle around the heart area on the surface of the chest. In this way, time delays and signal attenuations occur in only one detected signal, which is a combination of motion signals from different vibration sources in the heart, such as the four heart valves. Hence, Zanetti et al. [7] suggested using multiple accelerometers to reduce the influence of time delay and signal attenuation for the better detection of cardiac diseases. In the meantime, Lin et al. [8] identified six new feature points of cardiac activities, i.e., left ventricular lateral wall contraction peak velocity (LCV), septal wall contraction peak velocity (SCV), trans-aortic peak flow (AF), trans-pulmonary peak flow (PF), trans-mitral ventricular relaxation flow (MF_E_), and atrial contraction flow (MF_A_), which were not reported in any previous related works, with a novel multi-channel MCGs (or SCGs) system. With this system, more accurate timings of events have been identified among the previously found feature points. Some very important and meaningful cardia time intervals (CTIs) have then been calculated, such as the pre-ejection period (PEP), from ECG-Q to MCG-AO, and the left ventricular ejection time (LVET), from MCG-AO to MCG-AC. Reant et al. [9] proposed that the ratio of PEP to LVET, i.e., PEP/LVET, could also be an important index, known as the Contractility Coefficient (CC), for myo-cardiac functions. The key index used to justify the myo-cardiac functions, i.e., the left ventricular ejection fraction (LVEF), was also found to be strongly inversely correlated to the CC values in the study. Indeed, LVEF is an important clinical index for evaluating a human’s cardiac functions, especially for heart failure (HF) patients with a reduced ejection fraction (HFrEF).

Unlike the popular diagnosis instruments which are widely used clinically for cardiac health monitoring and examination, such as magnetic resonance imaging (MRI), computerized tomography scan (CT scan), and echocardiography (Echo), this technology, by combining ECG and MCG signals, can support long-term continuous cardiac health monitoring of patients [10]. To benefit from this technology ubiquitously in daily life for long-term and continuous monitoring of cardiac health monitoring, efforts towards implementing these technologies as wearable devices are desired. In the past years, several efforts have been made to study wearable devices or the body-sensing-network (BSN) for cardiac health monitoring [11,12,13,14], but all of them employed ECG-based technologies for the monitoring of cardiac health. The only two portable products based on MCG/SCG technology for cardiac health monitoring are the “Cardio Pro” from Heart Force Medical Inc., Vancouver, BC, Canada [15], and the one by M. Di Rienzo et al. [16]. However, both of them use a single channel MCG/SCG signal and still incorporate the drawbacks of the single-channel MCG/SCG technology.

Ballistocardiography (BCG) is another similar technology with SCG for cardiac activity monitoring [17]. The difference between the SCG and BCG signals is not simply a matter of nomenclature. Because the BCG measures whole body vibrations, the BCG signal is less influenced by local anatomical and sensor placement factors, and thus provides a better indication of hemodynamic information (e.g., CO) [18,19,20]. However, BCG signals cannot be measured as readily as SCG signals with wearable devices, but rather with weighing scales, tables, beds, or chairs—devices that can capture whole body movements. Moreover, a few works [21,22] proposed novel methods of measuring BCG signals using cameras or optical imagine and gained high-quality signals, but these restrict the measurement to fixed sites and hence are not able to benefit from the technologies anytime, anywhere, as the wearable system can provide. Recently, serval groups have demonstrated that BCG signals can also be measured using wearable accelerometers [23,24] and extensive data processing works have been implemented for data analysis [25]. Nevertheless, the waveform of the data was more distorted when more data processing techniques were used and this would make the analysis of event identification less accurate.

Furthermore, with the increased attention in research on wearable devices and smart clothing technologies, studies have also focused on trying to understand users’ perception of these smart technologies. Recent studies have used the technology acceptance model (TAM) [26], which is one of the most extensively used models to study an individual’s acceptance of information and communication technologies, to explain a user’s perception of wearable technology and smart clothes. For example, past studies [27,28,29] utilized the TAM to explain why users are more willing to adopt wearable technologies, such as smartwatches and wearable fitness products. Besides the original variables in the TAM, these studies also included other external constructs that were part of their research interests, such as visibility [27], affective quality, and mobility [28], as well as perceived health benefits [29]. However, even in recent studies that have sought to understand users’ perceptions of smart clothes, the participants were mostly generally healthier rather than patients with cardiovascular disease (CVD). Therefore, in this study, we have proposed an extension of the original TAM by adding external variables with the goal to explore the effects of external factors on attitudes, behavioral intentions, and the decision to use smart clothing technology among patients with CVD.

The goals of this work can be summarized as follows:

(1) CHAMP: Design of the Cardiac Health Assessment and Monitoring Platform (CHAMP) with multi-channel MCGs/ECG data acquisition, processing, and the wireless communication framework for mobile cardiac health monitoring.
(2) Smart Clothes: Implementing the framework as a wearable smart clothing for cardiac health monitoring.
(3) Methods & Assessment of Cardiac health monitoring: Development of an efficient mechanism for cardiac health assessment and prediction of the left ventricular ejection fraction (LVEF) for HF patients with detected multi-channel MCGs and ECG data.
(4) Usability study: Analysis of the extended technology acceptance model in understanding users’ behavioral intention to use a wearable cardiac health monitoring smart clothing system.

In this paper, the design and implementation of the wearable cardiac health monitoring and early warning system with multi-channel MCGs and ECG is described in Section 2, including: CHAMP; integration of CHAMP with textile-based technologies into smart clothing; methods of cardiac health assessment, especially for HF patients; and finally, the extended TAM model, the hypothesis proposition, and the details of data collection for this technology acceptance study. Section 3 presents the functional verification of the CHAMP, validations of the developed smart clothes, the analysis of output data from the system, and data correlation with myo-cardiac functions, as well as linkage to certain heart diseases, such as HFs, and the TAM results are also discussed. Finally, in Section 4, we conclude the entire paper. Some discussions and conclusions about the future direction of the work are drawn.

## 2. Materials and Methods

In this section, Section 2.1 describes the structure of CHAMP and its implementation for the synchronous multi-channel MCGs/ECG data acquisition, processing, and wireless data communication. The integration of the platform with textile-based technologies into wearable smart clothes is presented in Section 2.2. Section 2.3 introduces the CTIs that could be calculated from the system and how these can be used for the cardiac health assessment, such as how the LVEF can be derived from those CTIs and used for the cardiac health assessment of heart HF patients. Section 2.4 describes the extended TAM model, the hypothesis proposition, and the details of data collection for the usability and technology acceptance study.

### 2.1. CHAMP: Cardiac Health Assessment and Monitoring Platform

**Figure 1:**
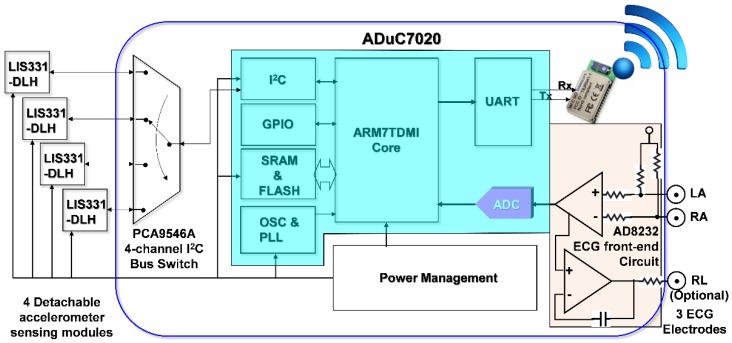
Block diagram of CHAMP (Cardiac Health Assessment and Monitoring Platform).

In order to simultaneously acquire data from multi-channel MCG units and a single channel ECG unit of the monitoring system, it is necessary to be able to read data from multiple accelerometers and digitized ECG data within a sampling period sequentially. To facilitate these requirements and considering mobility for support as wearable devices, a cardiac health assessment and monitoring platform, CHAMP, was designed and implemented. The block diagram of CHAMP is shown in Figure 1. The core architecture of CHAMP is a microcontroller to access four accelerometers through a four-channel I^2^C bus switch and digitized ECG signal from ECG front-end circuitry sequentially within one sampling period. Then, data can be calculated and filtered by the firmware in the microcontroller and the processed data are transmitted using UART protocol through the BlueTooth module. Detailed functions and selection of the components are discussed below.

#### 2.1.1. The Hardware Design of CHAMP

The main circuit of CHAMP is an embedded system with the microcontroller ADuC7020 from Analog Devices, Inc. (Norwood, MA, USA) as the core controller of this platform. The microcontroller has an internal crystal oscillator and can operate up to the core clock of 41.78 MHz, and the core is an ARM7TDMI core in 16-bit/32-bit RISC architecture with a 62 kB flash memory for program storage and 8 kB of SRAM for program execution. It includes multiple analog-to-digital converters (ADCs) providing a 12-bit resolution with a sampling rate up to 1 mega sampling per second (MSPS) on each channel. It also has built-in UART, I^2^C, and SPI on-chip peripherals and four general purpose timers. In the CHAMP design, an I^2^C interface is used to access multiple accelerometers, a UART peripheral is used to communicate with a Bluetooth module for wireless data transmission, an ADC is used to digitize the analog ECG signal so that it can be synchronized with multi-channel MCG data from accelerometers, and a timer is also set in the firmware to control the timing of event handling (data acquisition, processing, and transmission) for real-time data streaming to the receiver device.

Accelerometers used in this platform are LIS331DLH, from STMicroelectronics, Geneva, Switzerland. With the sampling rate of 400 Hz, these accelerometers are set at the sensing range of ±2 g at a 12-bit resolution, so that a 1 mg sensitivity (1 mg = 2^−10^ g, i.e., 1/1024 g, where g is the gravity force) can be achieved. Raw data acquired from each accelerometer are the acceleration along the *X*-, *Y*-, and *Z*-axis, but only the *Z*-axis acceleration data (in the direction perpendicular to the chest surface area) were processed and transmitted wirelessly. In this multi-channel MCGs/ECG system design, the four accelerometers used are the same components from the manufacture, and hence these all have the same I^2^C address. The use of a single channel of the I^2^C interface built in the microcontroller, ADuC7020, is not enough to identify these accelerometers separately. To be able to access these four accelerometers separately, a four-channel I^2^C bus switch, PCA9546A, from Texas Instruments (Dallas, TX, USA), is used and the specific bits in its control register are set to define which channel of the I^2^C interface to use. Therefore, a specific accelerometer can be accessed through that selected slave I^2^C interface.

In this design, an ECG measurement front-end circuit, AD8232, a fully integrated single-lead ECG front end chip which is capable of providing high signal gain (G = 100) with dc blocking capabilities, from Analog Devices, Inc., with surrounded discrete components (resistors and capacitors), is integrated. The ECG signal can be extremely noisy, and the AD8232 circuit can work as an op amp to obtain a clear ECG analog signal. A Lead I limb ECG measurement circuit is implemented. With a positive reference electrode attached to the left arm (LA) and negative reference electrode attached to the right arm (RA), it can obtain an optimal ECG waveform, configured with a 0.5 Hz high-pass filter followed by a 40 Hz low-pass filter. The RLD circuit that drives the third electrode, which is usually attached to the right leg (RL), is used to cancel the common-mode interference. However, it is optional and in our smart clothes integration, this input is not connected. Since CHAMP is integrated into smart clothes, the electrodes for the LA and RA can only be located on the upper trunk.

To provide the capability of wireless data transmission, a Class 1, Bluetooth (BT) 2.1+EDR module, RN-41 from Microchip Technology Inc. (Chandler, AZ, USA), is integrated into CHAMP. This BT module is operated in a serial port profile (SPP) and configured with a 115,200 buad-rate and none parity bit, 8 data bits, and 1 stop bit (N-8-1) standard. The UART interface is used for the data communication between the microcontroller and BT module. As a result, the platform can stream the data either through a RS-232 cable or BT module under the same firmware control.

**Figure 2:**
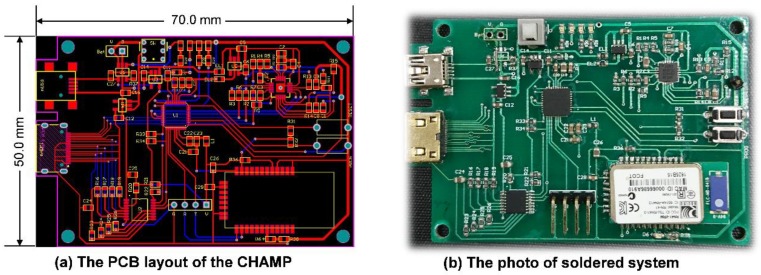
(**a**) The PCB layout of CHAMP; (**b**) the photo of the soldered system.

After finishing the schematic design of CHAMP, the PCB layout was completed with two metal layers with a size of 50 mm × 70 mm, as shown in Figure 2a. No special efforts were made to shrink the size of the PCB layout and hence there is still space to further reduce the size of the CHAMP PCB in future versions. Then, the PCB was fabricated with the FR4 panel and Figure 2b shows a photo of the actual system after all the components were soldered and the function of the board was tested. The total hardware cost of this platform, including the cost of PCB and all the components, is only around US$130. With larger quantities of hardware platforms made, a lower cost can be achieved.

#### 2.1.2. The Firmware Design of CHAMP

**Figure 3:**
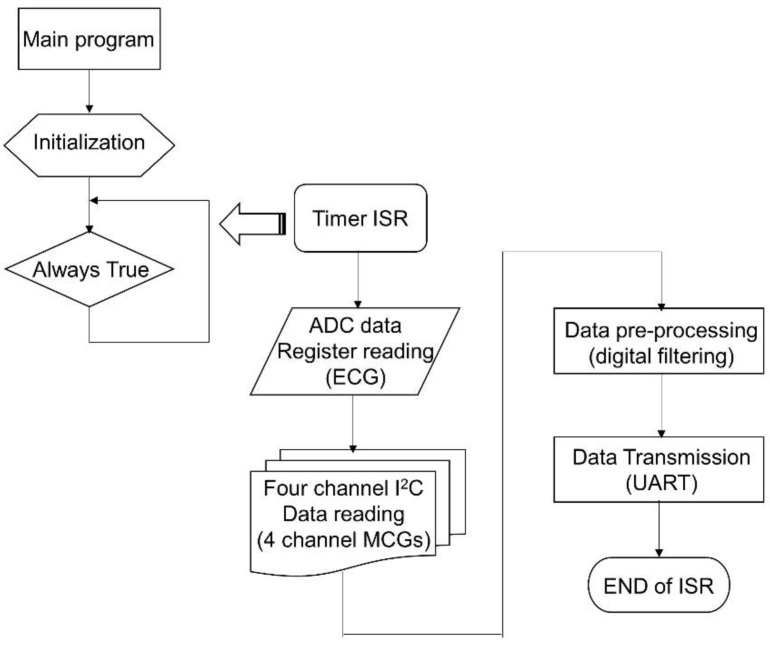
The flowchart of the firmware for CHAMP.

The firmware acts as the soul of CHAMP. Without proper implementation of the firmware, the functions of CHAMP would not perform as expected. Figure 3 shows the flowchart of the firmware, a simple interrupt driven program. Once the system is powered up, the firmware starts from its main program. In the main program, the system is initialized with proper configuration of the accelerometers (400 Hz sampling and output data rate, 0.5 Hz high-pass filter enabled), setting of the timer (every 2.5 ms), ADC configuration (400 SPS, 12-bit resolution), general purpose input-output (GPIO) configuration, UART initialization (115,200 bps, N-8-1 standard), and interrupt confirmation and enabling, etc. After the system has been initialized, the main program enters into an infinite loop waiting for the timer interrupt to occur. It is worth mentioning that the on-chip ADC is used to digitize the analog ECG signal from the ECG measurement front-end circuit. To save the power consumption of the microcontroller and to match the sampling rate with MCG signals, the sampling frequency of the ADC is set to use the timer signal.

The timer interrupt is set to generate interrupt signals to the microcontroller for every 2.5 ms, i.e., 400 Hz frequency. Once the microcontroller receives the timer interrupt, the timer interrupt service routine (ISR) is executed. In the timer ISR, it disables any further interrupt from occurring, and then reads the ADC output data register. After reading ECG data from ADC, it uses the I^2^C interface to go through the PCA9546A 4-channel I^2^C bus switch and to read each of the digital outputs of the four accelerometers one by one. After the ECG and four-channel MCG data are retrieved, the signal pre-processing task follows. In the signal pre-processing, digital filtering of a 50 Hz low-pass filter through the single ECG and four-channel MCG data is performed. Then, the output data after filtering are transmitted out of the system through the UART peripheral via the BT module. Before exiting the timer ISR, all interrupts are enabled again and wait for the occurrence of the next interrupt event.

**Figure 4:**
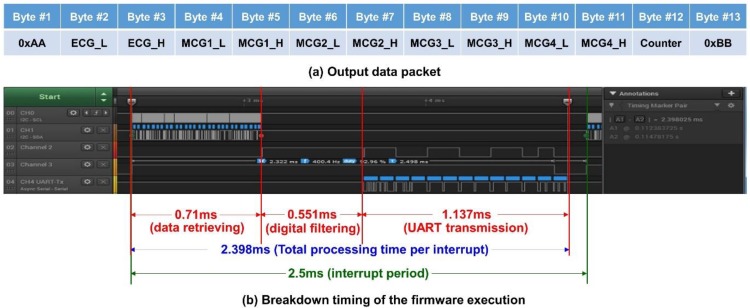
(**a**) The output data packet of the system; (**b**) the timing breakdown of the firmware execution.

Because the timing is critical for this kind of high data rate (400 Hz) real-time system, only *Z*-axis data from each accelerometer for the MCG signals are processed and transmitted. In this platform, 13 byte of data are transmitted from the system with UART protocol at 115,200 bps. The output data packet is shown in Figure 4a. The data packet contains the header byte, 0xAA, 2 byte of data for ECG, and the four accelerometers’ data (2 byte each), followed by an 8-bit counter value for the checking of continuity, and ends with a tail byte, 0xBB. The timing breakup of the firmware execution for the three major functional blocks, i.e., data retrieving, digital filtering, and UART transmission, is shown in Figure 4b, in a logic analyzer. With the microcontroller retrieving the ECG and 4 MCG data through I^2^C, it takes 0.71 ms for data preprocessing of digital filtering for a single ECG; for 4 ECG data, it takes 0.551 ms; and finally, sending out the filtered data through UART takes 1.137 ms. Therefore, the total execution time of the timer ISR is 2.398 ms, which is still less than the timer interrupt period of 2.5 ms (i.e., 400 Hz). From the measurement, it can be concluded that the system is actually performing real-time data acquisition, processing, and streaming wirelessly.

The raw data measured with accelerometers around the heart area on the chest surface can be extremely noisy. The signals have to be filtered for cleaner information and passed through the following processes. The ECG front-end circuit is already implemented with hardware high-pass and low-pass filters, so the major concerns are the MCG signals. The accelerometer used in the system is equipped with a built-in high-pass filter with certain cut-off frequency configurations. In the system, the accelerometer output data rate is set at 400 Hz and the built-in high-pass filter with a 1 Hz cut-off frequency is configured to block-out the dc value of acceleration due to the gravity and orientation of the accelerometer in use. To filter out the high frequency noise, the digital filtering process is desired for the raw MCG data. A low-pass FIR filter with a 50 Hz cut-off frequency and 60 dB signal drop-off at 60 Hz under a 400 Hz sampling frequency is designed with the FDATool in MATLAB^®^. With this FIR low-pass filtering design specification, a FIR digital filter with 79 taps is generated. With generated fixed-point coefficients and a filtering program implemented in C, the firmware incorporated with FIR digital filtering using fixed-point operations and a circular buffer mechanism is achieved within 0.551 ms of execution time for one ECG signal channel and four MCG signal channels. 

### 2.2. The Multi-Channel MCGs/ECG Monitoring Smart Clothes

**Figure 5:**
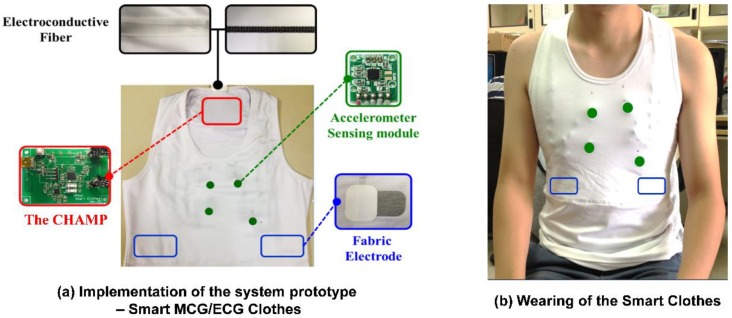
The prototype of multi-channel MCGs/ECG monitoring Smart Clothes.

To avail benefits from this technology anytime, anywhere, and for any movement in daily life for long-term and continuous monitoring of cardiac health monitoring, CHAMP has been integrated into a wearable system, i.e., multi-channel MCGs/ECG monitoring smart clothes. With our previous experience [30] and understanding of the advanced textile-based conductive techniques, we teamed up with AiQ, a Smart Clothing company, to integrate CHAMP and sensors into proper locations of smart clothes and had it fabricated as the prototype of the multi-channel MCGs/ECG monitoring smart clothes, as shown in Figure 5.

Figure 5a shows the integration of the previously discussed hardware platform and sensors, i.e., CHAMP, into smart clothes. The smart clothes were fabricated as a stretchable vest, so that it is skin-tight enough to collect steady and reliable MCG and ECG signals. Four accelerometer sensors were placed at proper locations, as in a previously reported study in [8], as the green dots marked in Figure 5a,b. The ECG electrodes were made with fabric electrodes, which can deliver more than 37 dB of SNR, for the collection of ECG signals, and the quality of ECG signals measured with these electrodes was also verified and is described in Section 3.1.1. The main circuit board of CHAMP is embedded at the location near the neck area, as shown in Figure 5a. It is connected to the accelerometer sensors and ECG electrodes with electro-conductive fiber for the data collection. A battery was also placed at the back of the main board to provide the working power. As a matter of fact, the only rigid parts of this smart clothing are the CHAMP board, battery, and four accelerometer sensor modules (10 mm × 7 mm each), and they weighed 70 g in total. The weight of the clothes is 245 g, including the rigid parts. Even so, from our usability study, patients still show a positive attitude towards using this system. Figure 5b shows the smart clothes worn by a human subject.

### 2.3. Cardiac Health Assessment for Heart Failures

**Figure 6:**
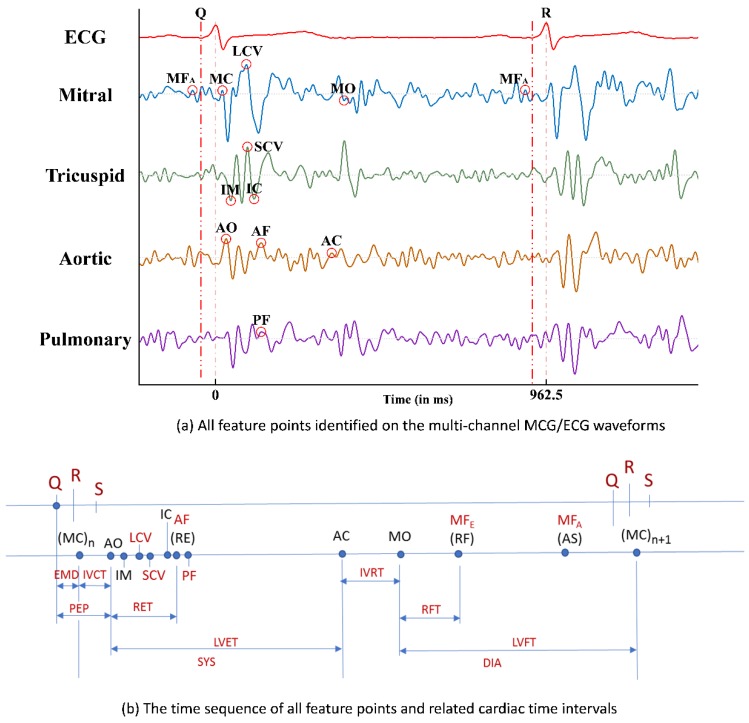
(**a**) All feature points identified on the multi-channel MCGs/ECG waveforms; (**b**) the time sequence of all feature points and related cardiac time intervals.

With the six newly identified feature points in the multi-channel MCG spectrum reported in Ref. [8] and the other feature points (FPs) which were identified and reported previously in Ref. [5], the time sequencing of these 15 feature points identified in the multi-channel MCGs/ECG data, along with the Q, R, and S points in the ECG signal, are plotted in Figure 6a. By calculating the time differences between certain FPs, some meaningful cardia time intervals (CTIs) can be obtained. For example, there are six CTIs related to the function of heart contraction, e.g., electro-mechanical delay (EMD) from the time point of ECG-Q to MCG-MC, iso-volumeric contraction time (IVCT) from MCG-MC to MCG-AO, pre-ejection period (PEP) from ECG-Q to MCG-AO, rapid ejection time (RET) from MCG-AO to MCG-AF (or RE), left ventricular ejection time (LVET) from MCG-AO to MCG-AC, and systole (SYS) from MCG-MC to MCG-AC. The time sequence of these feature points and the obtained CTIs are marked in Figure 6b. 

Clinically, the left ventricular ejection fraction (LVEF) is the key index to justify the myo-cardiac functions, especially for HF patients with a reduced ejection fraction (HFrEF). A higher LVEF value indicates healthier myo-cardiac functions. Among these CTIs, Buell [9] proposed that the ratio of PEP to LVET, i.e., PEP/LVET, could also be an important index for myo-cardiac functions, i.e., the Contractility Coefficient (CC), as in Equation (1), and LVEF was also found to be inversely correlated to the CC values in the study.

CC = PEP/LVET,(1)

Hence, with the related feature points identified from the multi-channel MCGs and ECG signal, and with those CTIs calculated accordingly, the CC value can be derived and thereafter, the correlated LVEF can be obtained as the assessment index for myo-cardiac functions, which is especially helpful for the cardiac health monitoring of HF patients.

### 2.4. Extended TAM Verification–Models, Hypothesis, and Data Collection

The TAM has been shown to successfully explain users’ perceptions of wearable technologies. In the TAM, perceived ease of use (PEOU) and perceived usefulness (PU) are two key psychological constructs that determine users’ attitudes toward the use of a technology or service. That is, if a technology is perceived as easy and useful for accomplishing a task, then users will have a more positive attitude toward the technology. Furthermore, a user’s attitude will later affect his/her intention to actually use the technology. Due to the explanatory ability of the TAM framework, the TAM has been consistently revised and validated in various fields of study. In this study, we extend the TAM to understand how other constructs, such as technology anxiety, perceived ubiquity, resistance to change, and benefit, would affect CVD patients’ perceptions of wearable smart clothing technology. As a result, we postulated the following hypotheses:

**Hypothesis** **1.** *Technology anxiety will be negatively associated with the perceived usefulness of a wearable cardiac health monitoring system among patients with cardiovascular disease.*

**Hypothesis** **2.** *Perceived ubiquity will be positively associated with the perceived usefulness of a wearable cardiac health monitoring system among patients with cardiovascular disease.*

**Hypothesis** **3.** *Perceived ubiquity will be positively associated with the perceived ease of use of a wearable cardiac health monitoring system among patients with cardiovascular disease.*

**Hypothesis** **4.** *Perceived ubiquity will be negatively associated with resistance to change with respect to using a wearable cardiac health monitoring system among patients with cardiovascular disease.*

**Hypothesis** **5.** *Perceived ubiquity will be positively associated with attitudes toward the use of a wearable cardiac health monitoring system among patients with cardiovascular disease.*

**Hypothesis** **6.** *Resistance to change will be negatively associated with the behavioral intention to use a wearable cardiac health monitoring system among patients with cardiovascular disease.*

**Hypothesis** **7.** *Benefit will be positively associated with the behavioral intention to use a wearable cardiac health monitoring system among patients with cardiovascular disease.*

**Hypothesis** **8.** *Perceived usefulness will be positively associated with attitudes toward the use of a wearable cardiac health monitoring system among patients with cardiovascular disease.*

**Hypothesis** **9.** *Perceived ease of use will be positively associated with the behavioral intention to use a wearable cardiac health monitoring system among patients with cardiovascular disease.*

**Hypothesis** **10.** *Attitude will be positively associated with the behavioral intention to use a wearable cardiac health monitoring system among patients with cardiovascular disease.*

In total, 48 participants who were older than 20 years and were either diagnosed as having HF with a left ventricular ejection fraction <40% or severe valvular heart disease agreed to participate in the study. Ethical approval was obtained from the Institutional Review Board (IRB) of Chang Gung Hospital, Taoyuan, Taiwan (104-8175B). Among total subjects, 77% of the participants were male and 23% were female. In terms of age, 8% were between 20 and 29 years; 8% were between 30 and 39 years; 19% were between 40 and 49 years; 25% were between 50 and 59 years; 23% were between 60 and 69 years; 8% were between 70 and 79 years; 6% were between 80 and 89 years; and 2% were older than 90 years. Regarding education, 10% had graduated from a graduate school; 35% had graduated from a university; 21% had completed high school as their highest level of education; 8% had completed junior high school as their highest level of education; and 25% had completed elementary school as their highest level of education.

When a participant arrived in the clinical room for this research survey, the researchers first explained the purpose and the procedure of the study explicitly. Then, the participants signed the consent form. During the experimental stage, the participants’ demographic data were collected. Subsequently, the researchers presented the developed wearable MCG-based cardiac health monitoring system to the participants so that they could actually feel the product and have a better sense of the texture and the functions of the wearable cardiac sensing technology. After completing the scenario presentation, the participant would then answer a technology acceptance questionnaire. The whole survey took approximately 30 min.

The technology acceptance questionnaire consisted of eight major sections that assessed technology anxiety, perceived ubiquity, resistance to change, benefit, perceived usefulness, perceived ease of use, attitude, and behavioral intention. All constructs were measured using five-point Likert-type scales, with 1 indicating strongly disagree and 5 indicating strongly agree. Each construct’s corresponding questionnaire was derived and modified from a variety of sources to reflect the characteristics of smart clothes. The measures for technology anxiety and resistance to change were adapted from Guo et al. [31]. The measure for perceived ubiquity was adapted from Hsiao and Tan [32]. The measure for benefit was adapted from Demiris et al. [33]. The measures for perceived usefulness and perceived ease of use were derived from Davis [26]. The measure of attitude originated from Fishbein and Ajzen [34] and Ajzen [35], and the measure of behavioral intention originated from Venkatesh et al. [36]. The data analysis included two stages: the measurement model and the path analysis. The measurement model was used to test the reliability and the validity of the constructs by conducting a confirmatory factor analysis. The path analysis was used to test the proposed research hypotheses. SPSS 22 and LISREL 8.7 were used to respectively perform the analyses of the measurement model and the path analysis.

## 3. Results and Discussions

### 3.1. Functional Verifications of CHAMP

The major functions of CHAMP are presented in this section. It verifies the ECG signal measurement from the in-system AD8232 ECG acquisition circuit and performs signal verification of digital filtering for both ECG and MCG signals.

#### 3.1.1. Verification of ECG Signal Measurement

**Figure 7:**
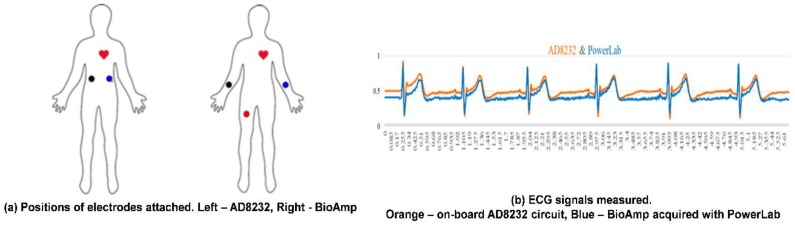
The verification of the on-board AD8232 ECG monitored signal. (**a**) Comparison of electrode positions; (**b**) ECG signal comparison.

The integration of the ECG monitoring circuit into CHAMP is a very important feature of the system. Therefore, the ECG signal measured from the on-board AD8232 ECG monitoring circuit is compared with the ECG signal measured from Bio Amplifier using commercially available electrodes simultaneously, as in the previous study [8], to confirm the correctness of the ECG measured signal from the on-board AD8232 circuit. As shown in Figure 7b, the timing of ECG signal feature points, i.e., P, Q, R, S, and T, measured from the on-board AD8232 circuit, is in-line with the one measured from Bio Amp and data acquired by PowerLab. In this comparison study, as shown in Figure 7a, not only the ECG signals are compared, and the positions of electrodes for AD8232 ECG measurement are attached on the upper trunk of the body instead of the hands and leg, which are the locations of electrodes attached in the conventional limb ECG measurement, such as the ECG measurement via Bio Amp in previous study. The comparison results concluded that the function and performance of the on-board AD8232 ECG monitoring circuit are reliable and the quality of ECG signals measured from fabric electrodes is about the same when compared with the commercial available electrodes.

#### 3.1.2. Verification for Digital Filtering Implementation

**Figure 8:**
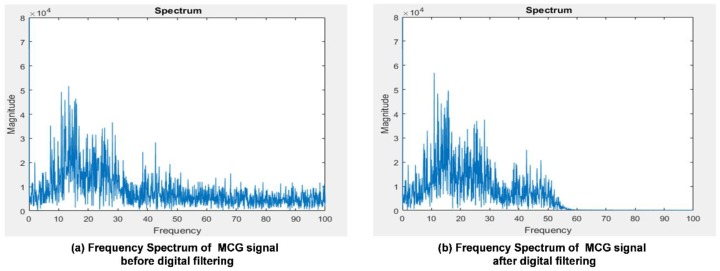
The frequency spectrum comparison of a single channel MCG signal before and after digital filtering in the gateway.

To validate the digital filter implementation, and to verify if the output of the filtered signals meets the filter design specification, the frequency spectrum comparison of a single MCG channel signal before and after the digital filtering is shown in Figure 8. Figure 8a shows the original frequency spectrum of an MCG signal before digital filtering is applied and Figure 8b depicts the frequency spectrum of that MCG signal after applying the digital filtering. From Figure 8b, it can be observed that the signal starts to drop-off from 50 Hz and the magnitude almost drops to 0 after 60 Hz compared with the original spectrum shown in Figure 8a.

**Figure 9:**
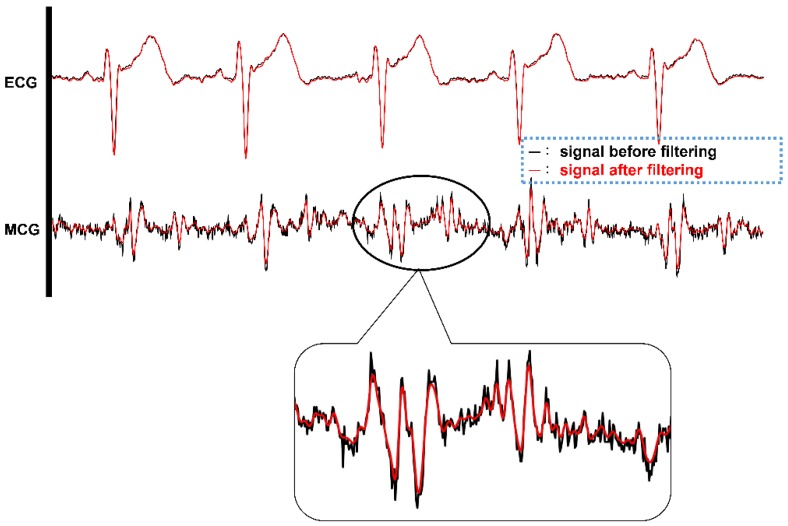
The timing signals of ECG and single channel MCG before (BLACK color) and after (RED color) digital filtering.

Even though there is a delay of 40 data samples (around 100 ms in this case) after digital filtering, the timing references to the ECG signal still remain the same since both the ECG signal and four-channel MCG signals go through the same digital filtering and suffer the same amount of delay. By aligning the ECG signal and one channel of the MCG signal in the time domain before and after the digital filtering, it is very clear to observe the effect of noise filtering, as shown in Figure 9. In Figure 9, the timing signal before filtering is shown in a black color and after filtering is shown in a red color. Clearly, from the zoom-in portion of the MCG signal, the red line is smoother, i.e., less affected by high frequency noise, after applying the digital filtering on the MCG signal. The ECG signal does not seem to vary a lot before and after digital filtering. This is because the data from the ECG signal for digital filtering were already filtered through the hardware low-pass filter implemented in the AD8232 circuit.

### 3.2. Validations of Multi-Channel MCGs and ECG Smart Monitoring Clothes

**Figure 10:**
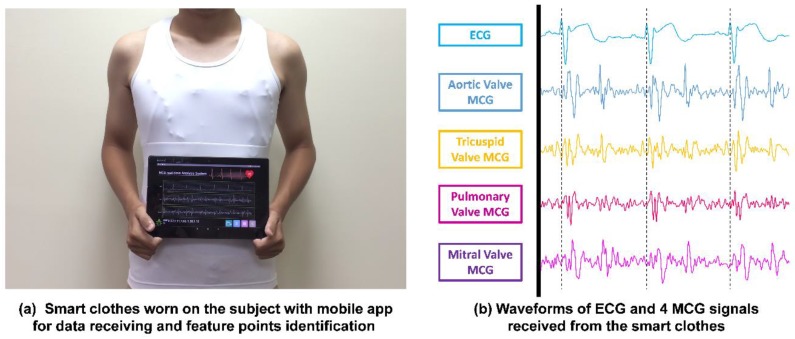
(**a**) Smart clothes worn by a subject with a mobile app for data receiving and auto identification of feature points; (**b**) the actual measured multi-channel MCGs/ECG signals plotted from the output data transmitted from the smart clothes.

Figure 10a shows the picture of a subject wearing the multi-channel MCGs/ECG smart monitoring clothes. The subject also holds an android-based tablet PC running a mobile app for obtaining data received from the smart clothes system wirelessly and performing the identification of feature points automatically. The feature points identified by the mobile app are the newly identified six feature points (FPs) reported in Ref. [8] and other feature points of cardiac activity events reported in Ref. [5]. The waveforms of the data received from these smart monitoring clothes through BlueTooth are shown in Figure 10b, i.e., one ECG waveform and four channels of MCG waveforms. Similar waveforms and features of the signals could be identified visually as in the previous study 8 and it validated the correctness of the multi-channel MCGs and signal-channel ECG measurement with the wireless transmission feature. The sampling rate of the accelerometer was set at 400 Hz. The signals seem to be nosier (lower SNR) than the ones shown in Ref. [24]; however, BCG signals were measured from the vibration of the body, which have bigger amplitudes compared with MCG signals that measure the tiny vibration signals in the chest area caused by cardiac activities. Also, a narrower bandwidth filter (0.5–25 Hz) was used in Ref. [24] and it is more likely to distort the signal waveforms compared to the original ones. Even though the MCG signals acquired from these smart clothes look nosier than the other related works, based on the time-window-based Morphological identification rules proposed previously by the comparison with echocardiography images, all the feature points could be identified accurately by the app, which has the identified rules implemented on the android-based tablet PC.

Taking the example of PEP and LVET calculation, the ECG-R, ECG-Q, MCG-MC, MCG-AO, and MCG-AC feature points have to be identified. According to the proposed time-window-based morphological identification rules, these feature points could be identified with the following rules.

- ECG-R: Use the So-and-Chan detection algorithm [37] to find the ECG-R point.
- ECG-Q: Find the first valley point, which defines the ECG-Q point, before the time instance of ECG-R point identified in step a.
- MCG-MC: On the MCG Mitral Valve (MV) channel, find the first peak right before the minimum point within the ECG-Q to ECG-R + 0.04 s time window. The peak defines the MCG-MC point.
- MCG-AO: On the MCG Aortic Valve (AV) channel, find the first peak right before the minimum point within the time instance of MCG-MC to ECG-R + 0.06 s time window. If no peak can be found, continue searching for the peak backward to ECG-Q. The peak defines the MCG-AO point.
- MCG-AC: On the MCG Tricuspid Valve (TV) channel, find the first peak, i.e., A point, right before the minimum point within ECG-R + 0.32 s to ECG-R + 0.5 s time window. On the MCG AV channel, find the first peak, which defines the MCG-AC point, backward from the time instance of A point.

Note that, when searching for the local valley points or peak points, if there is more than one data point with the same value, select the first data point from the searching direction as the local valley or local peak point. 

**Figure 11:**
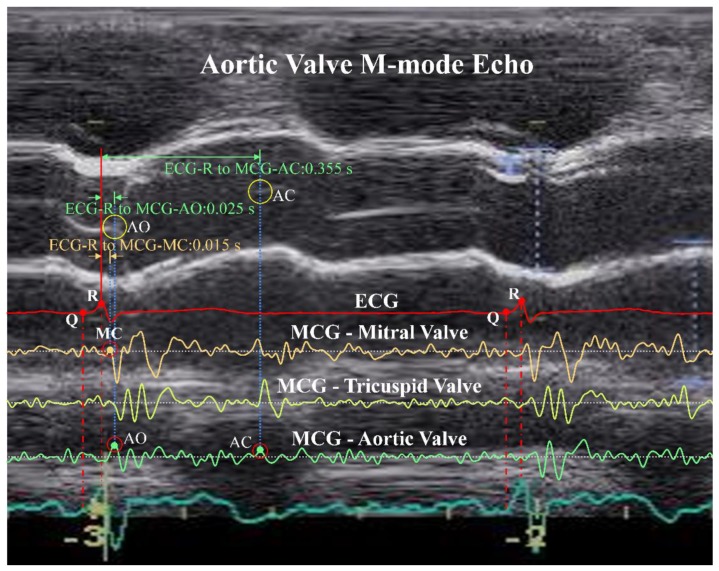
Confirmation of identified ECG-R, ECG-Q, MCG-MC, MCG-AO, and MCG-AC feature points with an Aortic Valve M-mode echocardiography image.

Among the identified feature points, i.e., ECG-R, ECG-Q, MCG-MC, MCG-AO, and MCG-AC, of eight randomly selected subjects, 10 continuous heart beat cycles from each of these subjects were chosen randomly to compare with their echocardiography image. Figure 11 illustrates the mapping of an echo image with measured ECG/MCG signals to confirm the accuracy of the identification results in one of the typical cycles. In the figure, ECG-R and ECG-Q were identified correctly on the ECG channel. Then, MCG-MC was identified on the MCG MV channel using the rule described in step c mentioned above and was confirmed with the Mitral Valve of the M-mode echo image, which is now shown in this figure. After that, the MCG-AO and MCG-AC were identified on the MCG AV channel according to the rules described in step d and e and were confirmed with the Aortic Valve M-mode echo image shown in the figure. The time intervals in the second cycle for ECG-R to MCG-MC, ECG-R to MCG-AO, and ECG-R to MCG-AC are also marked in the figure. All identified feature points were found matched with the events corresponding to their echocardiography images. The detailed identified time instances of the feature points compared with the inspection of echocardiography images are provided in the Supplementary Materials.

### 3.3. Data Analysis for Myo-Cardiac Function Interaction

**Figure 12:**
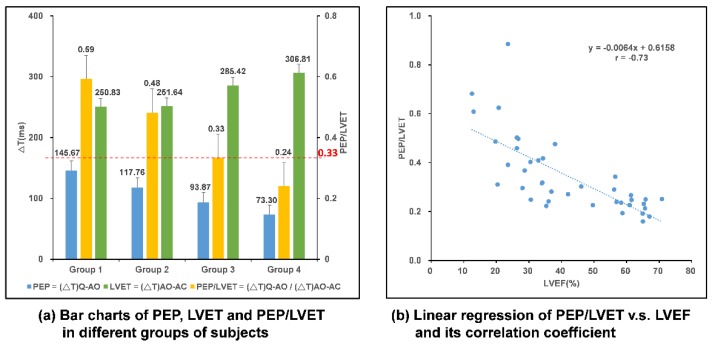
Analysis of data from multi-channel MCGs/ECG smart monitoring clothes and the myo-cardiac function interpretation. (**a**) Bar chart comparison in different groups of subjects; (**b**) linear regression of PEP/LVET, i.e., CC, v.s. LVEF.

To verify the correlation between CC and LVEF as proposed in Ref. [9], a study which was reviewed and approved by the institutional review board (IRB) of the Chang Gung Memorial Hospital, Taiwan R.O.C., was conducted. Twenty-five HF patients and 15 healthy subjects were enrolled in this study. Each subject wore the smart clothes for 30 min in a supine position and data were collected. These forty subjects were classified into four groups according to their LVEF, as suggested in Ref. [9], and the results are listed in Table 1. The bar charts of PEP, LVET, and PEP/LVET averages of the subjects in these four groups are plotted in Figure 12a and the linear regression of PEP/LVET, i.e., CC, v.s. LVEF, is plotted in Figure 12b. It is very clear that PEP/LVET is inversely proportional to the LVEF with a correlation coefficient of −0.73 and the *p* value is less than 0.001.

By conducting a regression analysis of the tested data, the LVEF value can be derived from the linear equation, as in Equation (2), with the CC value found and calculated from the multi-channel MCGs/ECG smart monitoring clothes.

PEP/LVET = −0.0064 * LVEF + 0.6158, and hence, LVEF = −156.25 * CC + 96.219(2)

According to the grouping of tested subjects shown in Table 1, the subjects in GP1-3 were HF patients, and for the CC value, a threshold value of 0.33 from the statistical analysis using receiver operating characteristic (ROC) curve, as shown by the red-dotted line in Figure 12a, could be set for justification of whether the subject is a HF patient or not. To further analyze the accuracy of the prediction of HF patients according to this threshold, FPs and CTIs from 50 heart beat cycles for HF patients and normal subjects were randomly retrieved from the data for blind testing. The outcomes of the prediction using that threshold are summarized in Table 2. With this analysis, the accuracy rate is 96%, and the positive predictivity rate is 94%, and a sensitivity of 98% and specificity of 94% are achieved. Among this testing, the true positive rate is 98%, false positive rate is 6%, true negative rate is 94%, and false negative rate is only 2%.

### 3.4. Analysis of the Extended TAM for the Smart Clothes Among Patients with CVD

To analyze the measurement model, Cronbach’s alpha and an item-total correlation were first obtained to measure the reliability of the individual items with respect to the corresponding construct variable. The recommended value for Cronbach’s alpha should be higher than 0.7, and the value for the item-total correlation is recommended to be higher than 0.3. The composite reliability was also measured, which tests the internal consistency within a construct. The value for composite reliability should be higher than 0.7. The Cronbach’s alpha value for all constructs, except for the perceived ease of use, surpassed the recommended value of 0.7. The Cronbach’s alpha for PEOU was 0.528, which is lower than 0.7. For the results of the item-total correlation, nearly all of the items had values greater than 0.3, although PEOU3 showed a value of 0.142, which is smaller than 0.3. The results for composite reliability showed that all constructs had values greater than 0.7, indicating a good internal consistency. Factor loading, which reflects how much a factor explains a variable, was also obtained by conducting a confirmatory factor analysis. Because we only included 48 participants in this study, which is considered to be a small sample size, MacCallum et al. [38] advocated that all items in a factor model should have communalities greater than 0.60 to justify the performance of a factor analysis with small sample sizes. The results showed that only PEOU1 had a factor loading of 0.418, while the other items all surpassed the value of 0.6. In terms of validity measures, we tested the construct validity, which included the convergent validity and the discriminant validity. Convergent validity refers to the degree of convergence of the items in the questionnaire in terms of one variable. For convergent validity to be statistically significant, the composite reliability should be higher than the recommended value of 0.7, and the average variance extracted (AVE) of construct variables should be higher than 0.5. Composite reliability has been examined previously, and the results for the AVE showed that only PEOU was smaller than 0.5, while the other constructs had values higher than 0.5. On the other hand, discriminant validity examines the degree to which two constructs that are supposed to be unrelated are distinguishable. To measure discriminant validity, the square root of the AVE of the measured construct should be larger than the correlation coefficient of the other dimensions. The square root of the average change of the construct was larger than the correlation coefficients of the other constructs. In addition to technology anxiety, the other constructs all showed mean values higher than 3. For technology anxiety, the mean value was 2.49, indicating that the participants did not have strong technology anxiety toward smart clothes. In short, the measurement model showed strong results in terms of both reliability and validity. However, perceived ease of use did not meet some of the recommended values in the measurement model.

We conducted a path analysis to test the 10 research hypotheses among eight constructs. The path coefficients and the statistical measurements are shown in Table 3. Eight out of ten hypotheses showed significant results, and two hypotheses were invalid. Hypothesis 1 hypothesized that by using a wearable cardiac health monitoring system, patients with CVD would feel that technology anxiety and perceived usefulness were negatively correlated. The path analysis results showed that *γ*1 = −0.39 (*t* = −3.80, *p* < 0.001). However, the mean value of technology anxiety showed that participants did not perceive the wearable system as anxiety-inducing. Therefore, Hypothesis 1 was not supported. Hypothesis 2 hypothesized that by using a wearable cardiac health monitoring system, patients with CVD would feel that perceived ubiquity and perceived usefulness were positively correlated. The path analysis results showed that *γ*2 = 0.62 (*t* = 6.07, *p* < 0.001). Therefore, Hypothesis 2 was supported. Hypothesis 3 hypothesized that by using a wearable cardiac health monitoring system, patients with CVD would feel that perceived ubiquity and perceived ease of use were positively correlated. The path analysis results showed that *γ*3 = 0.54 (*t* = 4.22, *p* < 0.001). Therefore, Hypothesis 3 was supported. Hypothesis 4 hypothesized that by using a wearable cardiac health monitoring system, patients with CVD would feel that perceived ubiquity and resistance to change were negatively correlated. The path analysis results showed that *γ*4 = −0.39 (*t* = −2.86, *p* < 0.01). Therefore, Hypothesis 4 was supported. Hypothesis 5 hypothesized that by using a wearable cardiac health monitoring system, patients with CVD would feel that perceived ubiquity and attitude were positively correlated. The path analysis results showed that *γ*5 = 0.28 (*t* = 2.13, *p* < 0.05). Therefore, Hypothesis 5 was supported. Hypothesis 6 hypothesized that by using a wearable cardiac health monitoring system, patients with CVD would feel that resistance to change and behavioral intention were negatively correlated. The path analysis results showed that *γ*6 = −0.39 (*t* = −3.35, *p* < 0.01). Therefore, Hypothesis 6 was supported. Hypothesis 7 hypothesized that by using a wearable cardiac health monitoring system, patients with CVD would feel that benefit and behavioral intention were positively correlated. The path analysis results showed that *γ*7 = 0.36 (*t* = 2.99, *p*< 0.01). Therefore, Hypothesis 7 was supported. Hypothesis 8 hypothesized that by using a wearable cardiac health monitoring system, patients with CVD would feel that perceived usefulness and attitude were positively correlated. The path analysis results showed that *γ*8 = 0.49 (*t* = 3.65, *p* < 0.05). Therefore, Hypothesis 8 was supported. Hypothesis 9 hypothesized that by using a wearable cardiac health monitoring system, patients with CVD would feel that perceived ease of use and behavioral intention were positively correlated. The path analysis results showed that *γ*9 = −0.20 (*t* = −1.69, *p* > 0.05). Therefore, Hypothesis 9 was not supported. Hypothesis 10 hypothesized that by using a wearable cardiac health monitoring system, patients with CVD would feel that attitude and behavioral intention were positively correlated. The path analysis results showed that *γ*10 = −0.22 (*t* = 1.73, *p* > 0.05). Therefore, Hypothesis 10 was not supported. Overall, seven hypotheses were supported, and three were invalid. The fit indices of the path analysis are listed in Table 4. All of the fit indices met the recommended values, indicating that the results of the path analysis are appropriate.

## 4. Conclusions

In this study, the realization of a wearable cardiac health monitoring and myo-cardiac function interpretation smart clothes with multi-channel MCGs and ECG measurement is presented. To acquire the digital data from multiple accelerometers and to convert the analog data of the ECG signal simultaneously, as well as to provide the capability of wireless data transmission, a platform, i.e., CHAMP, has been designed and implemented. It provides the mobility and capability for real-time data analysis and disease detections, so it is perfectly suitable to integrate the platform into smart clothes.

This work translated the concept of a multi-channel MCG system into a wearable smart clothes for cardiac health monitoring and myo-cardiac function interpretation system, which then can be applied for home-based or clinical usages to detect certain cardiovascular diseases, such as HFs. After the smart clothes delivered continuous real-time multi-channel MCGs/ECG data, data analysis for myo-cardiac function interpretation of this wearable system was validated. A mobile application software has been developed to receive the data from the smart clothes, identify feature points, and calculate CTIs, CC, and LVEF. Also, the relationship between CC and LVEF has been verified in this study. In the system, with the data received from the smart clothes, CC values are calculated and LVEF values are derived from the analyzed data. A highly accurate rate of prediction for HF patients with this system could be achieved. Hence, the developed smart clothes with multi-channel MCGs and ECG for cardiac health monitoring can be applied for long-term and continuous monitoring of myo-cardiac functions.

The usability study of the wearable system is also verified. We extended the technology acceptance model and investigated perceptions of the developed wearable MCG-based cardiac health monitoring system among potential users with CVD. The results of TAM analysis indicated that perceived ubiquity is a crucial construct that can affect participants’ perception of intention to use a wearable cardiac sensing technology, such as smart clothes. The perceived benefit of the newly designed wearable system increases participants’ willingness to adopt the smart technology. Furthermore, the perceived usefulness of the wearable MCG-based cardiac health monitoring system showed a positive correlation with the participants’ attitude toward it. The results of our hypothesized model contribute to the original TAM and the existing research on understanding users’ perceptions of wearable sensing technologies in general. In the future, several features of the system can be extended, for example, the application of the system for the prediction of other cardiovascular diseases, such as valvular heart diseases (VHDs), and also the risk assessment of certain heart diseases with expert systems (or machine learning algorithm), cloud computing, and trend analysis for early prediction, etc. Finally, this system can be applied to detect cardiovascular diseases, in particular HF and VHD, for patients and individual users who are self-conscious about their heart health.

In short, unique smart clothes for cardiac health monitoring system is designed and implemented. This is the first wearable system incorporated with multi-channel MCGs and ECG measurement technology which is capable of the long-term and continuous monitoring of cardiac health of the subjects in their daily life. In this work, a mobile application which receives data from the smart clothes, identifies the feature points automatically, and calculates CTIs for deriving the cardiac health-related indices, such as CC and LVEF, in real-time has also been developed. It can help to predict the abnormality of cardiac functions, such as the HFs, for the subjects wearing the clothes, with an accuracy rate of up to 96%. Moreover, the usability study of the smart clothes with proposed extended TAM indicates that people, especially CVD patients, show a positive attitude toward using this wearable MCG-based cardiac health monitoring and early warning system.

## Figures and Tables

**Table 1:** Statistics of four groups of subjects and their PEP, LVET, PEP/LVET, and LVEF values.

Groups (LVEF Range)	PEP (ms)	LVET (ms)	PEP/LVET	LVEF (%)
GP1 (<20%)	145.66 ± 11.91	250.83 ± 45.32	0.59 ± 0.10	15.07 ± 4.02
GP2 (21% < LVEF < 30%)	117.76 ± 37.40	251.64 ± 27.42	0.48 ± 0.18	24.91 ± 3.02
GP3 (31% < LVEF < 40%)	93.87 ± 27.84	285.42 ± 51.36	0.33 ± 0.09	34.26 ± 2.47
GP4 (>41%)	73.3 ± 13.47	306.81 ± 43.44	0.24 ± 0.05	59.57 ± 7.58

**Table 2:** Accuracy analysis for the prediction of HF patients.

	Predicted Abnormal	Predicted Normal	Total
Actual Abnormal	49	1	50
Actual Normal	3	47	50
Total	52	48	100

**Table 3:** Summary of the hypothesis results.

	IV	→	DV	Standardized Regression Coefficient	*T*-Value	*p*-Value	Support
Hypothesis 1	TA	→	PU	−0.39	−3.80	***	No
Hypothesis 2	PB	→	PU	0.62	6.07	***	Yes
Hypothesis 3	PB	→	PEOU	0.54	4.22	***	Yes
Hypothesis 4	PB	→	RC	−0.39	−2.86	**	Yes
Hypothesis 5	PB	→	AT	0.28	2.13	*	Yes
Hypothesis 6	RC	→	BI	−0.39	−3.35	**	Yes
Hypothesis 7	BF	→	BI	0.36	2.99	**	Yes
Hypothesis 8	PU	→	AT	0.49	3.65	*	Yes
Hypothesis 9	PEOU	→	BI	−0.20	−1.69		No
Hypothesis 10	AT	→	BI	0.22	1.73		No

*** *p* < 0.001, ** *p* < 0.01, * *p* < 0.05.

**Table 4:** Fit indices for the path analysis.

Measures	Recommended Criteria	Path analysis
*χ*^2^/df	<3.0	0.7275
GFI	>0.8	0.94
AGFI	>0.8	0.86
NFI	>0.9	0.94
NNFI	>0.9	1.04
CFI	>0.9	1.00
RMSEA	<0.08	0.000

## Supplementary Materials

The following are available online at http://www.mdpi.com/1424-8220/18/10/3538/s1.
